# Relevance of different prior knowledge sources for inferring gene interaction networks

**DOI:** 10.3389/fgene.2014.00177

**Published:** 2014-06-24

**Authors:** Catharina Olsen, Gianluca Bontempi, Frank Emmert-Streib, John Quackenbush, Benjamin Haibe-Kains

**Affiliations:** ^1^Machine Learning Group (MLG), Université Libre de Bruxelles (ULB)Brussels, Belgium; ^2^Interuniversity Institute of Bioinformatics Brussels ULB-VUBBrussels, Belgium; ^3^Computational Biology and Machine Learning Laboratory, Center for Cancer Research and Cell Biology, Queen's University BelfastBelfast, UK; ^4^Department of Biostatistics and Computational Biology, Dana-Farber Cancer Institute, Harvard School of Public HealthBoston, MA, USA; ^5^Department of Cancer Biology, Dana-Farber Cancer InstituteBoston, MA, USA; ^6^Bioinformatics and Computational Genomics, Princess Margaret Cancer Centre, University Health NetworkToronto, ON, Canada; ^7^Medical Biophysics Department, University of TorontoToronto, ON, Canada

**Keywords:** prior knowledge, validation, colon cancer, knockdown, network inference

## Abstract

When inferring networks from high-throughput genomic data, one of the main challenges is the subsequent validation of these networks. In the best case scenario, the true network is partially known from previous research results published in structured databases or research articles. Traditionally, inferred networks are validated against these known interactions. Whenever the recovery rate is gauged to be high enough, subsequent high scoring but unknown inferred interactions are deemed good candidates for further experimental validation. Therefore such validation framework strongly depends on the quantity and quality of published interactions and presents serious pitfalls: (1) availability of these known interactions for the studied problem might be sparse; (2) quantitatively comparing different inference algorithms is not trivial; and (3) the use of these known interactions for validation prevents their integration in the inference procedure. The latter is particularly relevant as it has recently been showed that integration of priors during network inference significantly improves the quality of inferred networks. To overcome these problems when validating inferred networks, we recently proposed a data-driven validation framework based on single gene knock-down experiments. Using this framework, we were able to demonstrate the benefits of integrating prior knowledge and expression data. In this paper we used this framework to assess the quality of different sources of prior knowledge on their own and in combination with different genomic data sets in colorectal cancer. We observed that most prior sources lead to significant *F*-scores. Furthermore, their integration with genomic data leads to a significant increase in *F*-scores, especially for priors extracted from full text PubMed articles, known co-expression modules and genetic interactions. Lastly, we observed that the results are consistent for three different data sets: experimental knock-down data and two human tumor data sets.

## 1. Introduction

Whilst it is now widely accepted that cellular processes are in general not only governed by single genes but instead also by networks of interacting genes (Barabási and Oltvai, 2004), there is no gold-standard for validating these biological networks (Yngvadottir et al., 2009; Fernald et al., 2011). However, as network inference is increasingly used in biomedical research such as drug discovery or disease classification (Barabási et al., 2011), also the subsequent validation needs to be revisited. The most commonly used approach consists in comparing the inferred network to known interactions stored in biological databases and research articles (Altay et al., 2013). However, this approach has three major drawbacks: Firstly, these interactions are rarely complete, secondly they might not be appropriate for the studied problem and lastly, their quality has not yet been evaluated.

An alternative use for this prior knowledge is its integration into the network inference algorithms in order to improve the quality of inferred networks. Indeed, we and others showed that the combination of data and prior knowledge significantly improves the quality of networks compared to networks inferred from data only (Djebbari and Quackenbush, 2008; Mukherjee and Speed, 2008; Olsen et al., 2014). However, if prior knowledge is used to improve the inference process its subsequent use in the quality assessment would dramatically increase the risk of overfitting.

Recently, we proposed a purely data-driven approach relying on experimental perturbation data to identify the set of relevant genes for a given problem (Olsen et al., 2014). This validation framework not only provides the possibility to compare different inference algorithms but furthermore allows us to independently assess different sources of prior knowledge by themselves and in combination with expression data.

In this follow-up paper to Olsen et al. (2014), we use the proposed validation framework to evaluate the quality of a variety of prior sources, both in combination with different publicly available tumor data sets and by themselves. We retrieved the prior knowledge using the two web applications *Predictive Networks* (Haibe-Kains et al., 2012b) and *GeneMANIA* (Mostafavi et al., 2008), for a total of eight different sources. After the assessment of the different prior sources' quality, we infer networks using three different microarray data sets: experimental knock-down data from cell line experiments and two publicly available human tumor data sets. We quantitatively assess their quality through the estimation of *F*-scores, a well established quality metrics in network inference.

We observe that most prior sources lead to significant *F*-scores. Their integration with genomic data leads to a significant increase in *F*-scores, especially for priors extracted from full text PubMed articles, known co-expression modules and genetic interactions. We also observe that the results are consistent for three different data sets: experimental knock-down data and two human tumor data sets. Furthermore, we observe that combining different sources can be beneficial compared to using a single prior source.

## 2. Materials and methods

### 2.1. Method—validation of inferred networks

**Figure 1:**
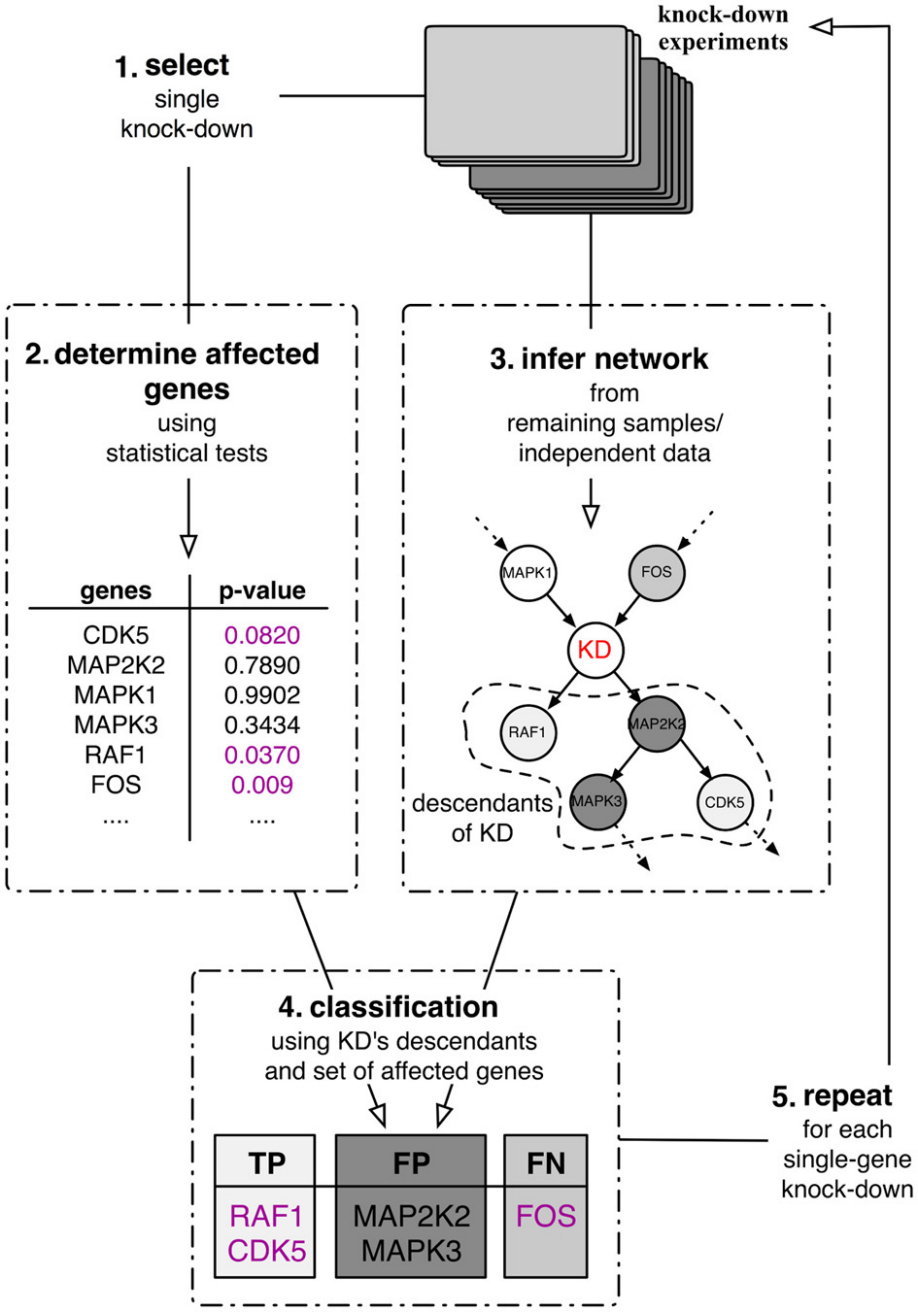
**Quantitative validation framework for network inference**. The framework relies on a set of single-gene knock-down experiments in a leave-one-out cross-validation scheme.

The best case scenario in most real-world application is partial knowledge of the true, data-generating network. Therefore, the assessment of any inferred network cannot depend on this knowledge alone. As an alternative, we proposed a purely data-driven validation framework proposed in Olsen et al. (2014). This validation framework depends on the availability of experimental intervention data such as knock-down experiments. This type of data allows us, for each knock-down experiment separately, to statistically evaluate whether or not a gene in the data set was significantly affected by the experiment. In this case, this relation should be reflected in any inferred network in the sense that the affected gene can be found downstream of the knocked down gene. This in turn then allows us to quantitatively assess the quality of inferred gene interaction networks by computing quality measures such as precision, recall or *F*-score (Sokolova et al., 2006). The outline of the framework is depicted in Figure 1. Suppose that a number of single gene knock-down experiments were carried out. Then one can use these experiments in a five step procedure:

1. Select a single knock-down and all corresponding replicates from the collection.
2. Use these samples to determine the set of genes that were significantly affected by the perturbation experiments by means of statistical tests.
3. Use the remaining independent samples to infer a directed network.
4. Classify the knock-down's descendants (in the inferred network) into true positives, false positives and false negatives with respect to the affected genes identified in step 2. The descendants of a node in the network are defined to be the set of its children and grandchildren.
5. Repeat steps 1–4 until all perturbations have been used to assess the network's local predictive power.

In Olsen et al. (2014), a network was inferred from the samples not related to the single knock-down experiment (step 3). However, in the same article it was shown that these knock-down samples from cell line experiments can be used for validation not only in such a cross-validation scheme but also for networks inferred from independent tumor samples, which demonstrates the generalizability of our validation approach.

The classification of the nodes in the network (step 4) follows the rationale that statistically significantly affected genes should be found in a directed network downstream of the perturbed gene, its descendants (Figure 1). Therefore all genes in the set of descendants which are significantly affected by the perturbation can be classified as true positives (TP) and all significantly affected genes that are inferred outside of the set of descendants as false negatives (FN). Genes that are part of the descendants in the inferred network but are not significantly affected by the perturbation are then false positives (FP).

This classification then allows us to compute the *F*-score, the harmonic average of precision and recall

(1) $$F=\frac{2\cdot TP}{2\cdot TP+FP+FN}\in [0,1],$$

where *F* = 0 corresponds to no correctly identified affected genes and *F* = 1 corresponds to perfect classification.

To control for the density of the network and thus guaranteeing that the *F*-scores are meaningful, we generated 1000 random networks. Each random network is obtained from the inferred network by shuffling the genes in this network.

### 2.2. Material—data

Throughout this study, we use the perturbation data described in Olsen et al. (2014), which are publicly available in the NCBI Gene Expression Omnibus (GEO) repository (Barrett et al., 2005), under accession number GSE53091. The samples of this data set consist of eight single gene knock-downs, namely CDK5, HRAS, MAP2K1, MAP2K2, MAPK1, MAPK3, NGFR, and RAF1. These genes belong to the RAS signaling pathway which has been showed to play a key role in colorectal cancer (Zenonos and Kyprianou, 2013). The knockdown experiments were performed in two colon cancer cell lines, SW480 and SW620 (NCBI Gene Expression Omnibus (GEO) repository (Barrett et al., 2005) accession number GSE53091). For each knock-down, six biological replicates were obtained together with controls in both cell lines, in total 125 samples. The data set furthermore consists of the 339 variables over expressing RAS as identified in Bild et al. (2005) and used in Olsen et al. (2014).

For each of the knocked down genes we identify the significantly affected genes by comparing the expression of genes in control versus those of the knock-down experiments with a Wilcoxon Rank Sum test, using a false discovery rate (FDR, Benjamini and Hochberg, 1995) <10% as a threshold for statistical significance. In Table 1 we present the number of affected genes for each of the knock-down experiments.

**Table 1:** **Number of genes significantly affected by KD (out of 339 genes) based on gene expression data with FDR <10%**.

**KD**	**CDK5**	**HRAS**	**MAP2K1**	**MAP2K2**
Number of affected genes	73	122	33	38
	**MAPK1**	**MAPK3**	**NGFR**	**RAF1**
	117	59	99	61

We will use two publicly available tumor cancer data sets (expO, 2009; Jorissen et al., 2010) to infer the networks. The first data set (*expO*) contains 292 human tumor samples and is accessible from GEO under accession number GSE2109. The second (*jorissen*) data contains 290 samples and is accessible from GEO under accession number GSE14333.

### 2.3. Material—sources of prior knowledge

Possible sources of prior knowledge are manifold and include published articles, interactions stored in biological databases or similarity of gene expression values, also referred to as gene co-expression, from published data sets. To efficiently access this information a number of different tools have been implemented including *GeneMANIA* (Mostafavi et al., 2008) and Predictive Networks (Haibe-Kains et al., 2012a). The former allows to upload a set of genes and returns a network of the known interactions distinguishable by source (Table 2) whereas the latter uses text mining to retrieve known interactions from PubMed abstracts and furthermore queries structured biological databases. Both tools allow to download the interactions as flat text files, which enables further use of these priors into advanced genomic analyses such as gene interaction network inference.

**Table 2:** **Specifications of prior knowledge retrieval tools: *GeneMANIA* (GM) and *Predictive Networks* (PN)**.

**Tool**	**Source**		**# interactions**
PN	PubMed and databases	(PN)	419
	Co-expr	(GM2)	2760
	Co-local	(GM3)	292
	Genetic	(GM4)	1546
GM	Pathway	(GM5)	100
	Physical	(GM6)	38
	Predicted	(GM7)	29
	Shared	(GM8)	199

Here we will use the complete prior set retrieved by *Predictive Networks* (PN) and priors separated by source from *GeneMANIA*. The different number of known interactions identified by each tool and source are presented in Table 2. These can be roughly grouped into three categories: (1) Co-expression and genetic with > 1000 interactions; (2) PN and co-local, pathway and shared with 100 to ~400 interactions; and (3) physical and predicted with <50 interactions.

## 3. Results

In this section we use the proposed validation framework (Figure 1) to independently assess the quality of the different priors retrieved with *Predictive Networks* and *GeneMANIA* (Table 2) in isolation and in combination with three different genomic data sets.

We use the inference procedure introduced in Haibe-Kains et al. (2012a,b) which is a two-step procedure implemented in the R/Bioconductor package *predictionet*. The first step is a feature selection step based on the minimum redundancy, maximum relevance (mRMR, Ding and Peng, 2005; Meyer et al., 2007) criterion whose robustness is improved by the integration of prior knowledge. The subsequent step is an arc orientation procedure using a criterion based on interaction information (McGill, 1954) in which prior integration is used to help orient the edges which could not be oriented from the genomic data. Given the central role of priors in predictionet, we implemented a hyperparameter, referred to as prior weight (*w*), enabling users to tune their confidence in the prior knowledge incorporated into the network inference procedure. Prior weight *w* can take value from 0 to 1; low *w* stands for low confidence in prior data. Note that *w* = 0 forces *predictionet* to ignore priors (only genomic data are taken into account), while predictionet with *w* = 1 will infer networks solely based on prior information, therefore ignoring genomic data.

We use each of the three different data sets (kd, *expO* and *jorissen*) to build networks integrating the different prior knowledge sources with different prior weights *w* ∈ {0, 0.25, 0.5, 0.75, 0.95, 1}. The validation is then carried out for each of the eight knocked down genes. We thus obtain eight *F*-scores, one for the descendants of each KD. These *F*-scores are then further assessed by comparing them to *F*-scores of 1000 random networks.

### 3.1. Prior information only

**Figure 2:**
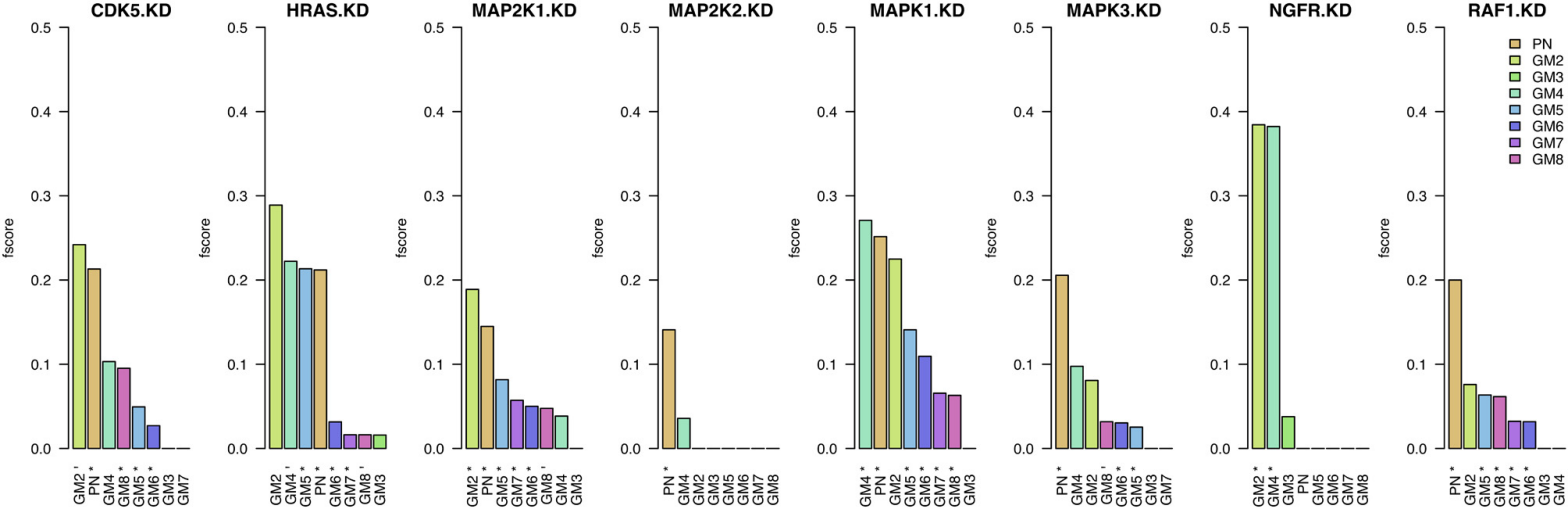
**Results when inferring networks with *predictionet* using only prior knowledge (*w* = 1)**. The height of each bar corresponds to the obtained *F*-score, colored by prior source. The *x*-axis specifies the prior source and includes ^*^ if the *F*-score is significant with *p*-value <0.05 and ^−^ for *p*-values < 0.1.

The first step in the assessment of the different prior sources' quality is the evaluation of the networks inferred using only these sources (prior weight *w* = 1). In Figure 2, we present the results in terms of *F*-scores and significance compared to random networks. When assessing this figure with respect to the number of significant results obtained by each prior source, we can observe that PN performs best with seven out of eight significant results. The next best prior sources are GM6 and GM5 with six significant KDs. With the exception of GM3, all prior sources have at least two significant results. Furthermore, the *F*-scores obtained using prior source PN are amongst the highest values for all KDs except NGFR. On the contrary, GM6 obtains six significant KDs but the *F*-scores are all below those obtained by PN.

Assessing the prior sources' performance with respect to the eight knock-downs, it can be observed that some KDs are in general better predicted than others. Whilst most prior sources are able to obtain significant results for HRAS, MAP2K1, MAPK1, and RAF1, significant results for half the prior sources for CDK5 and MAPK3 they struggle to provide meaningful information for inference of gene interactions in the context of colorectal cancer with the remaining two knock-downs (MAP2K2 and NGFR).

### 3.2. Combination of data and prior information

In this section we assess the networks inferred from genomic data (KD data in cross-validation; Figure 1) and prior knowledge with equal weight (*w* = 0.5). In a first analysis, we compare these *F*-scores to those obtained when inferring networks from data only (*w* = 0) and from prior knowledge only (*w* = 1). A statistical test (Wilcoxon rank test) shows that the combination of data and prior significantly improves the networks (*p*-values <0.05) compared to data only (Supplementary Table 1) and prior only (Supplementary Table 1, with exception of GM2).

**Figure 3:**
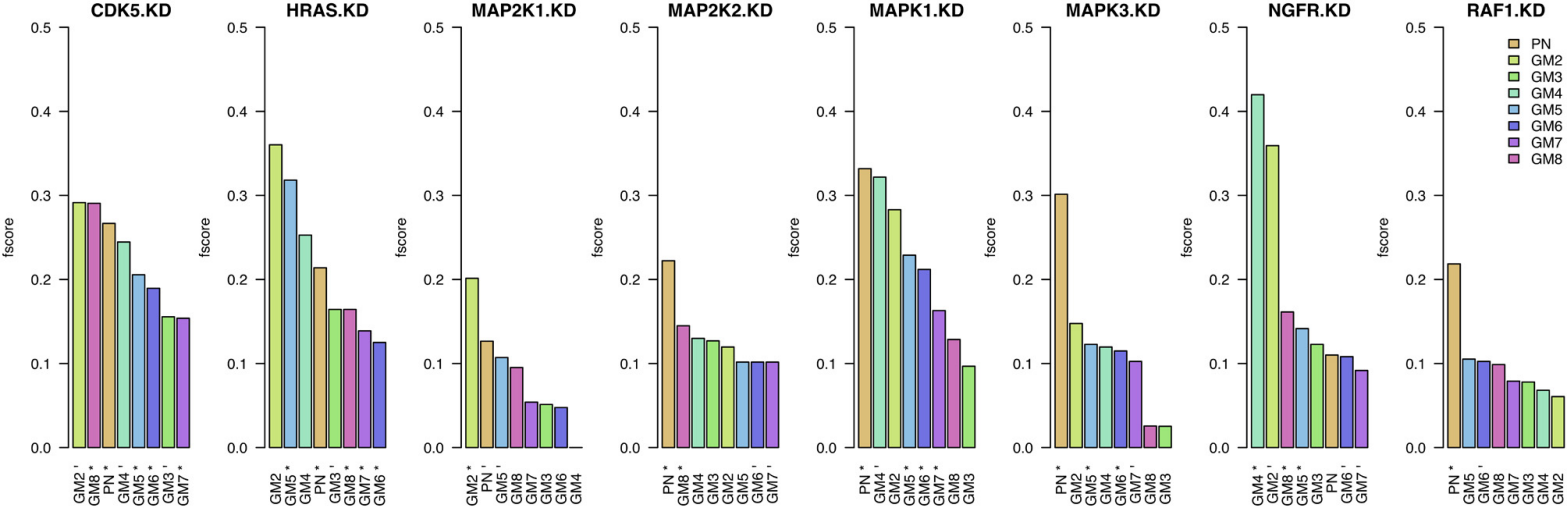
**Results when inferring networks with *predictionet* using data and prior knowledge (*w* = 0.5)**. The height of each bar corresponds to the obtained *F*-score, colored by prior source. The *x*-axis specifies the prior source and includes ^*^ if the *F*-score is significant with *p*-value <0.05 and ^−^ for *p*-values < 0.1.

In Figure 3, we present these *F*-scores for each knock-down and for each of the eight prior sources. For each knock-down, the results are ordered by *F*-score values, starting with the best result and color-coded by prior source. The best prior source for four out of the eight knock-downs in PN: MAP2K2, MAPK1, MAPK3, and RAF1. The second highest number of best knock-downs is reached by GM2: CDK5, HRAS, and MAP2K1. The best prior source for NGFR is GM4. On the contrary, the performance of GM3, GM6, and GM7 prior sources is amongst the lowest.

### 3.3. Most consistent prior source across three different data sets

In this section, we will show that the results presented in the previous section for the KD data also hold true when the networks are inferred in combination with the two human tumor data sets. In Table 3, we present the prior source that yielded the highest *F*-score for each of the eight knock-downs (prior weight *w* = 0.5). This table summarized the results in Supplementary Figures 9 and 10.

**Table 3:** **Best single prior source across three large colorectal cancer data sets (kd for knock-down experiments in colorectal cancer cell lines, *expO* and *jorissen* for large human colon tumor data) when combined with microarray gene expression data (prior weight *w* = 0.5)**.

**KD**	**KD data**	**expO**	**Jorissen**
CDK5	GM2	PN	PN
HRAS	GM2	GM4	GM2
MAP2K1	GM2	GM2	GM2
MAP2K2	PN	PN	GM7
MAPK1	PN	PN	PN
MAPK3	PN	PN	PN
NGFR	GM4	GM4	GM4
RAF1	PN	GM8	PN

The main observation is that the best prior source is consistent for all three data sets for four of the eight knock-downs: MAP2K1, MAPK1, MAPK3, and NGFR. For the remaining four knock-downs, the best prior source is consistent for two out of the three data sets: PN for CDK5, MAP2K2, and RAF1 and GM2 for HRAS.

### 3.4. Combining different prior sources

**Figure 4:**
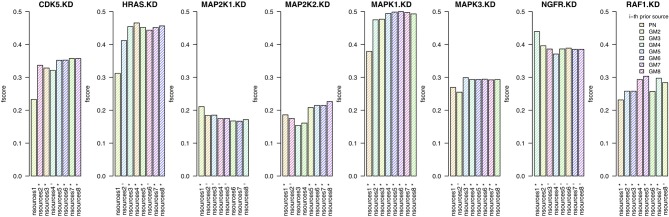
**Results when inferring networks with *predictionet* using *expO* data and prior knowledge (*w* = 0.5)**. The height of each bar corresponds to the obtained *F*-score, colored by which prior source was added. The *x*-axis specifies the prior source and includes ^*^ if the *F*-score is significant with *p*-value < 0.05 and ^−^ for *p*-values < 0.1.

In this section we investigate whether the combination of prior sources (from a single prior source upto all eight sources) is beneficial to the quality of the inferred networks. For each knock-down, we infer a network using the best prior source, then we add the second best, etc. (Figure 3). We test this procedure on the two independent human tumor data sets *expO* and *jorissen*, the corresponding results are presented in Figure 4 and Supplementary Figure 11, respectively.

When combining *expO* data and with an increasing number of prior sources, the results are better than those obtained using only one source for six out of the eight KDs. For the other two, namely MAP2K1 and NGFR, we have already observed in section 3.1 that most prior sources are not informative. The number of prior sources that need to be combined to obtain the highest significant *F*-scores depends on the knock-down and range between three and eight. It is therefore not only important to determine whether prior sources are relevant by themselves but also which combination of sources will lead to the best results. Similar observations can be made for the *jorissen* data set (Supplementary Figure 11).

## 4. Discussion

Using the quantitative validation framework we recently introduced in Olsen et al. (2014), we assessed the relevance of different sources of prior information for the inference of large gene interaction networks from high-throughput gene expression data sets. Our results suggest that most prior sources, which include known interactions extracted from research articles, genetic and physical interactions, co-expression and pathway databases yield significant networks in colorectal cancer when used in isolation. Furthermore, concurring with our previous results, we demonstrated that the vast majority of prior sources significantly improves the inference of gene interaction networks when combined with microarray gene expression data.

In our case study we showed that priors extracted from the *Predictive Networks* web application and the co-expressions reported in *GeneMANIA* are the most relevant prior sources in colorectal cancer as they yield the best networks in our validation study. We also showed that these results are consistent across three data sets, composed of a set of knock-down experiments in colorectal cancer cell lines and large collections of human colon tumor samples.

As expected, the quality of inferred gene interaction networks is not uniform over the network topology. For the eight genes we knocked down to investigate their effects in colorectal cancer cell lines, we were able to infer statistically significant subnetworks for most, but not all of them. For instance, we observed that the effects of NGFR, and MAP2K2 knock-downs are particularly difficult to model. Interestingly, genetic interactions and co-expression prior data enabled to build high quality networks for NGFR, which suggests that priors extracted from diverse sources are highly complementary.

Our study supports the use of prior information into network inference and we are now working on improving methods to extract high-quality, context-specific prior information, as well as developing novel approaches to integrate these priors to generate better large-scale gene interaction networks. A second aspect that requires further development is the implementation of tools to better combine different prior sources with the hope to significantly improve the local quality of large biological networks.

### Conflict of interest statement

The authors declare that the research was conducted in the absence of any commercial or financial relationships that could be construed as a potential conflict of interest.
