# Supplementary material for: Relevance of different prior knowledge sources for inferring gene interaction networks
Source: Front Genet. 2014 Jun 24;5:177. doi: 10.3389/fgene.2014.00177 (PMC4067568; doi:10.3389/fgene.2014.00177)
Supplement: Supplementary file 1 [file DataSheet1.PDF]

Frontiers in Genetics (Bioinformatics and Computational Biology)

Supplementary material: Relevance of different prior knowledge sources for inferring gene interaction networks

Catharina Olsen [1,2], Gianluca Bontempi [1,2], Frank Emmert-Streib [3], John Quackenbush [4,5] and Benjamin Haibe-Kains [6]

[1] *Machine Learning Group, Université Libre de Bruxelles, Brussels, Belgium*
[2] *Interuniversity Institute of Bioinformatics Brussels ULB-VUB, La Plaine Campus, Brussels, Belgium*
[3] *Computational Biology and Machine Learning Laboratory, Center for Cancer Research and Cell Biology, Queen's University Belfast, Belfast, UK*
[4] *Department of Biostatistics and Computational Biology, Dana-Farber Cancer Institute,and Department of Biostatistics, Harvard School of Public Health, Boston, MA, USA*
[5] *Department of Cancer Biology, Dana-Farber Cancer Institute, Boston, MA, USA*
[6] *Bioinformatics and Computational Genomics, Princess Margaret Cancer Centre, University Health Network, Toronto, Ontario, Canada*

Correspondence*:
Benjamin Haibe-Kains
Bioinformatics and Computational Genomics, Princess Margaret Research, Ontario Cancer Institute, University Health Network, Toronto, Ontario, Canada, bhaibeka@uhnresearch.ca

Quantitative Assessment and Validation of Network Inference Methods in Bioinformatics

1 FULL REPRODUCIBILITY OF THE ANALYSIS RESULTS

We will describe how to fully reproduce the result figures and tables reported in the main manuscript. We automated the analysis pipeline so that minimal manual interaction is required to reproduce our results. To do this, one must simply:

1. Set up the software environment
2. Run the R scripts

1.1 SET UP THE SOFTWARE ENVIRONMENT

We developed and tested our analysis pipeline using R running on linux and Mac OSX platforms.

To mimic our software environment the following R packages should be installed:

- R version 2.15.0 (2012-03-30), `x86_64-unknown-linux-gnu`
- Base packages: stats, graphics, grDevices, utils, datasets, methods, base
- Other packages: e1071_1.6, class_7.3-3, predictionet_1.1.8, catnet_1.13.2, igraph_0.5.5-4
- Loaded via a namespace (and not attached): graph_1.32.0, MASS_7.3-17, penalized_0.9-37, RBGL_1.30.1, splines_2.15.0, survival_2.37-4, tools_2.15.0

All these packages are available on CRAN[1] or Bioconductor[2].

Uncompress the archive provided as **Supplementary data** accompanying the manuscript[3] This should create a directory named `VALIDATION` on the file system containing the following files:

- `script.R` Script containing the definitions of all functions required for the analysis pipeline.
- `VALIDATION_pipeline.R` The implementation of the validation pipeline that generates the results and accompanying figures and tables.

Download the necessary data files from `http://www.ulb.ac.be//di/map/colsen/NetInfPriors/` and save them in a folder called `new_data` within the `VALIDATION` folder. All the files required to run the automated analysis pipeline are now in place.

1.2 RUN THE R SCRIPTS

Open a terminal window and go to the `VALIDATION` directory. You can easily run the analysis pipeline either in batch mode or in a R session.

To run the full pipeline in batch mode, simply type the following command:

```
R CMD BATCH VALIDATION_pipeline.R Rout &
```

The progress of the pipeline could be monitored using the following command:

[1] http://cran.r-project.org

[2] http://www.bioconductor.org

[3] The code is also available on GitHub: `https://github.com/bhaibeka/NetInfPriors`

```
tail -f Rout
```

To run the full analysis pipeline in an R session, simply type the following command:

```
source("VALIDATION_pipeline.R")
```

Key messages will be displayed to monitor the progress of the analysis.

The analysis pipeline was developed so that all intermediate analysis results are saved in the directories `saveres_kd`, `saveres_expo` and `saveres_jorissen` for the three data sets respectively. Therefore, in case of interruption, the pipeline will restart where it stopped. The main results will be stored in the directory `results`.

2 TABLE

Prior source	p-value	
	$w = 0.5$ vs $w = 0$	$w = 0.5$ vs $w = 1$
PN	0.0039	0.0117
GM2	0.0112	0.7266
GM3	0.0039	0.0391
GM4	0.0039	0.0039
GM5	0.0112	0.0273
GM6	0.0112	0.0039
GM7	0.0112	0.0078
GM8	0.0173	0.0078

Table 1. Results of paired Wilcoxon rank tests: comparing the networks inferred from data and prior (prior weight $w = 0.5$) with (i) data only network (first column) and (ii) prior only networks (second column).

3 DETAILS F-SCORES RANDOM NETWORKS

3.1 PRIOR SOURCE: PN

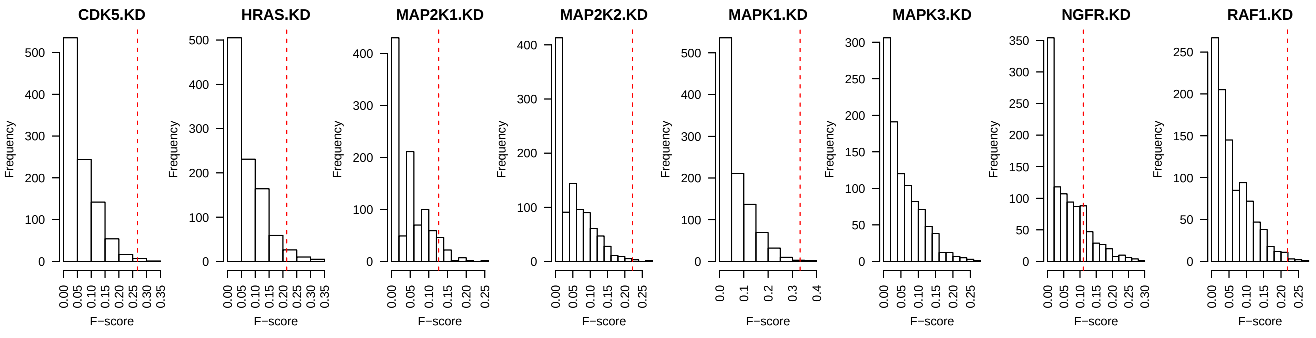

(a) Fscores random KD

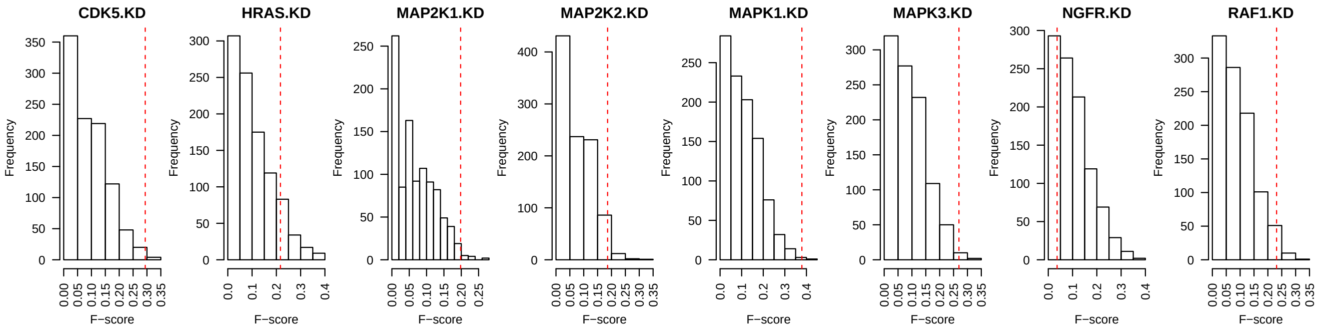

(b) Fscores random expO

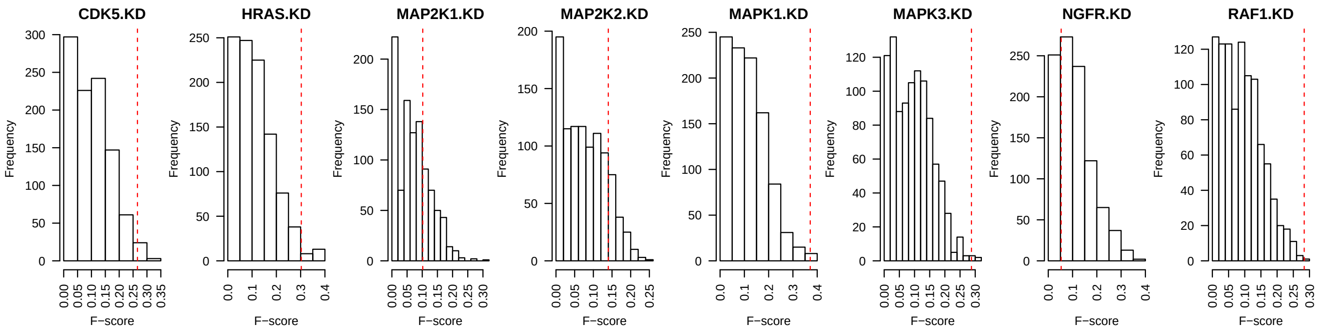

(c) Fscores random jorissen

Figure 1. Each subplot is the histogram of the obtained F-scores for the 1000 random networks. The red line represents the F-score obtained using *predictionet* for the prior source PN, data set and prior weight $w = 0.5$.

3.2 PRIOR SOURCE: GM2

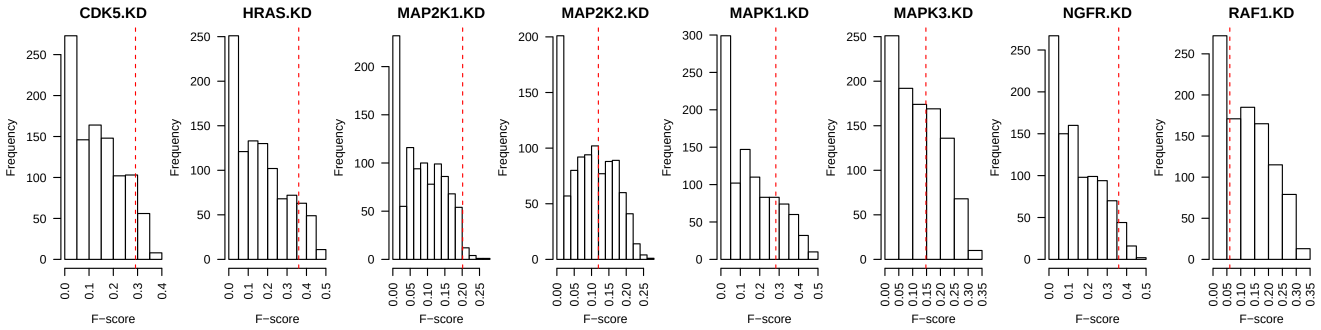

(a) Fscores random KD

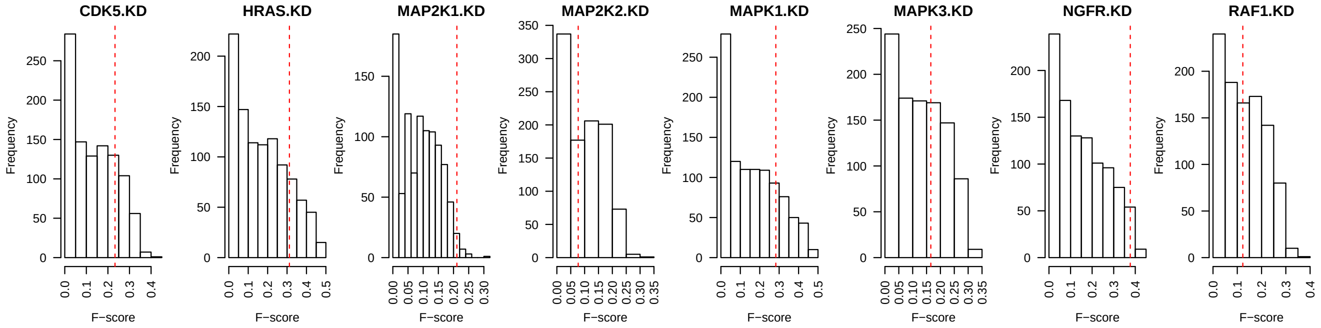

(b) Fscores random expO

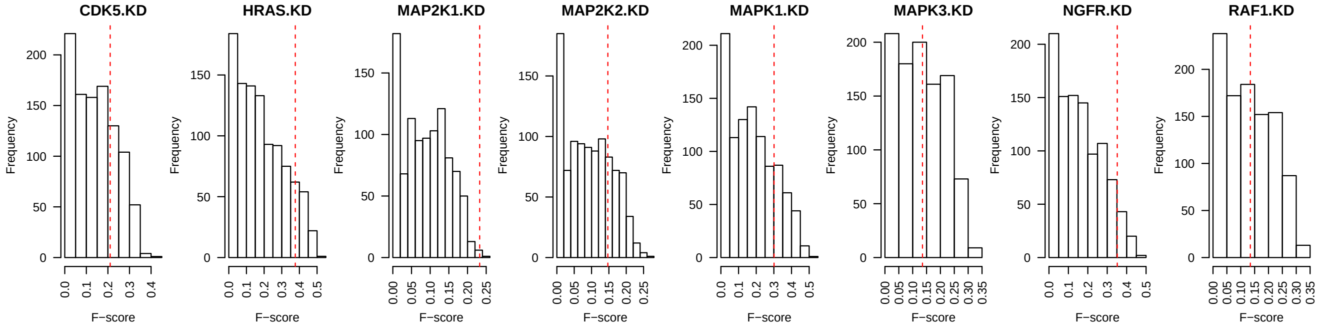

(c) Fscores random jorissen

Figure 2. Each subplot is the histogram of the obtained F-scores for the 1000 random networks. The red line represents the F-score obtained using *predictionet* for the prior source GM2, data set and prior weight $w = 0.5$.

3.3 PRIOR SOURCE: GM3

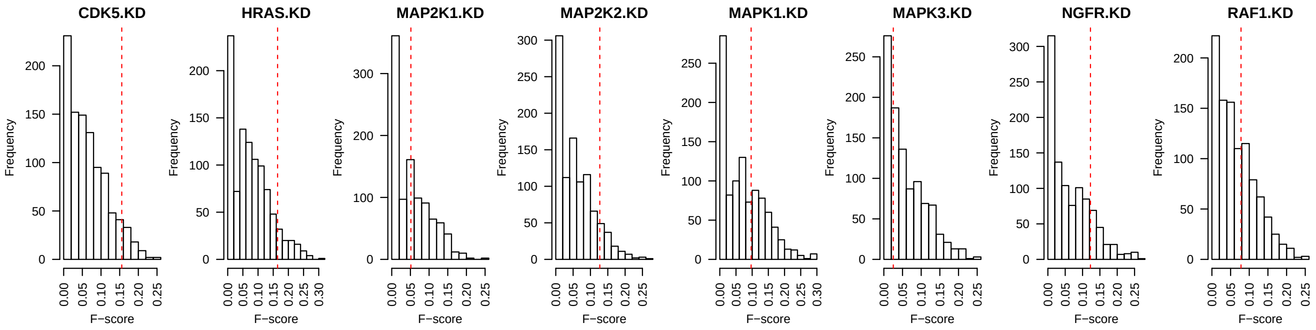

(a) Fscores random KD

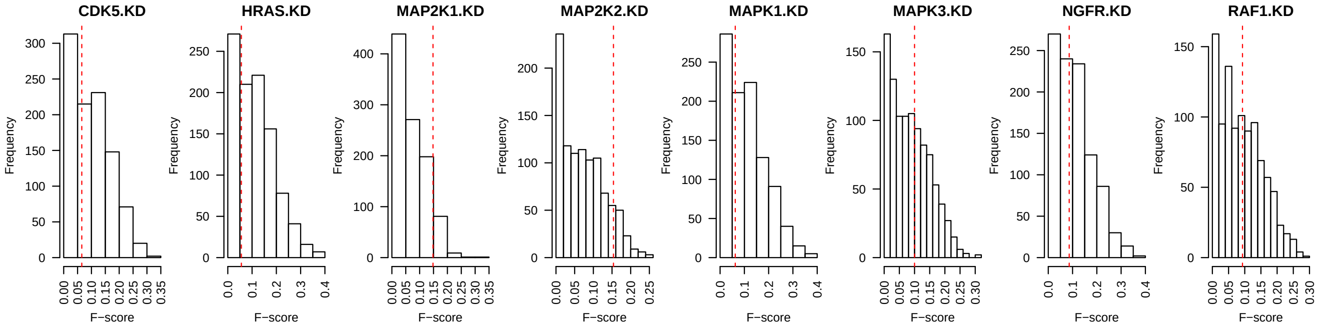

(b) Fscores random expO

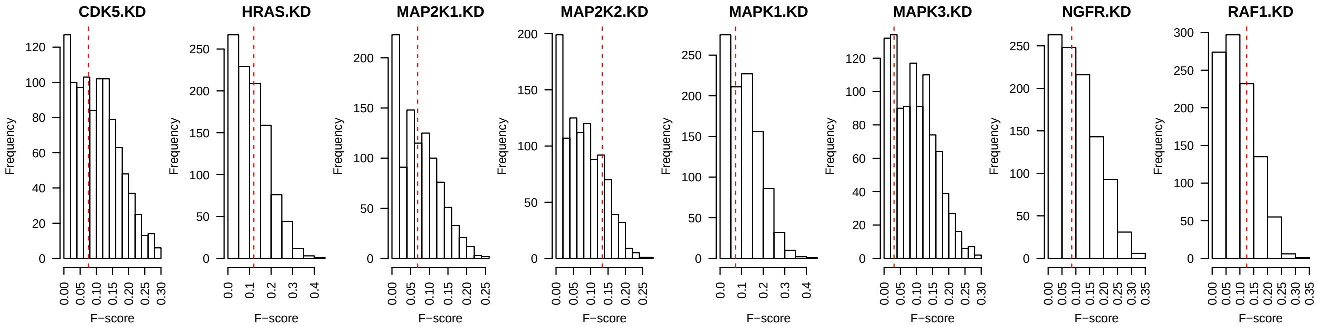

(c) Fscores random jorissen

Figure 3. Each subplot is the histogram of the obtained F-scores for the 1000 random networks. The red line represents the F-score obtained using *predictionet* for the prior source GM3, data set and prior weight $w = 0.5$.

3.4 PRIOR SOURCE: GM4

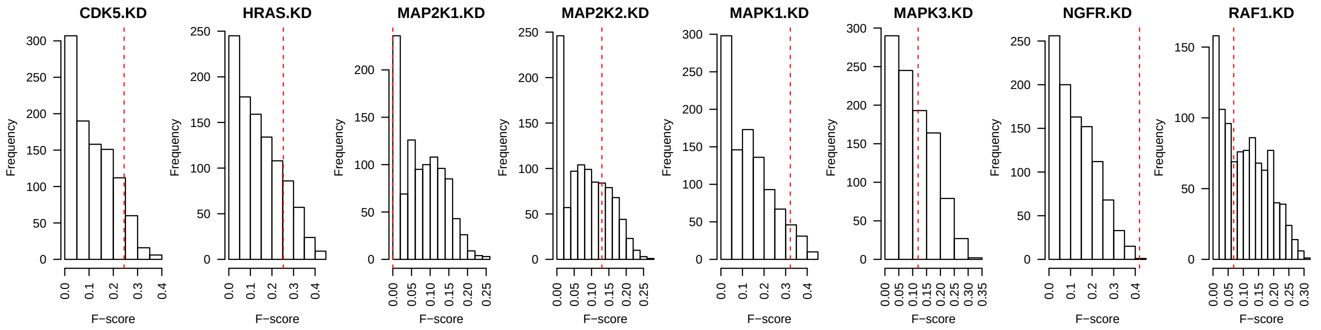

(a) Fscores random KD

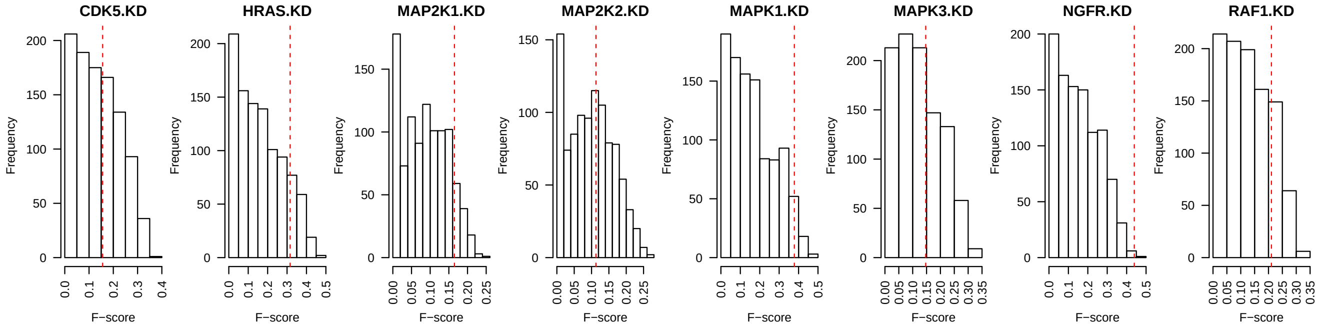

(b) Fscores random expO

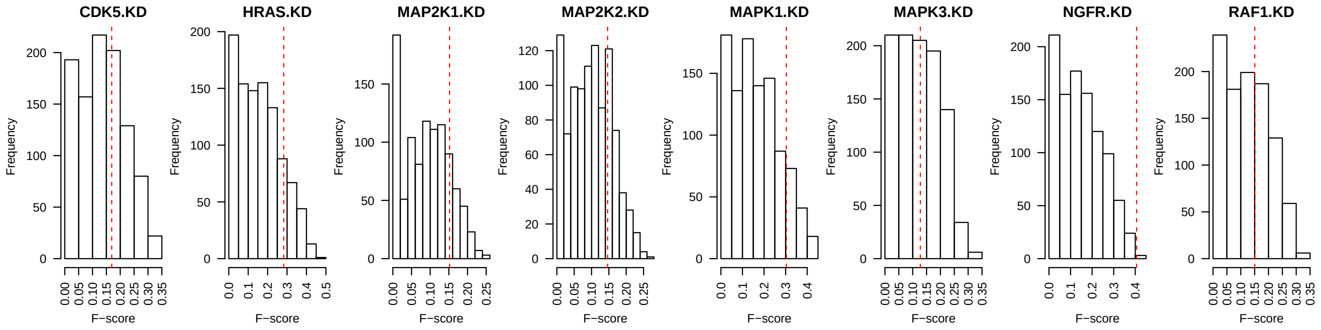

(c) Fscores random jorissen

Figure 4. Each subplot is the histogram of the obtained F-scores for the 1000 random networks. The red line represents the F-score obtained using *predictionet* for the prior source GM4, data set and prior weight $w = 0.5$.

3.5 PRIOR SOURCE: GM5

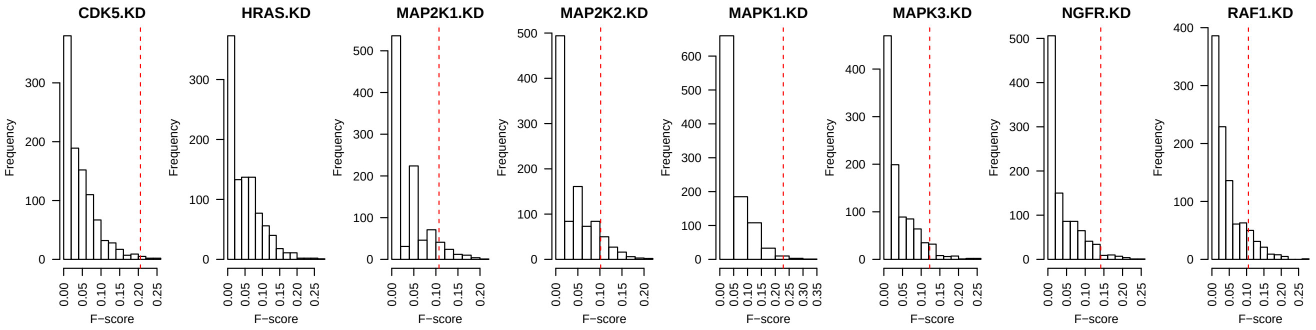

(a) Fscores random KD

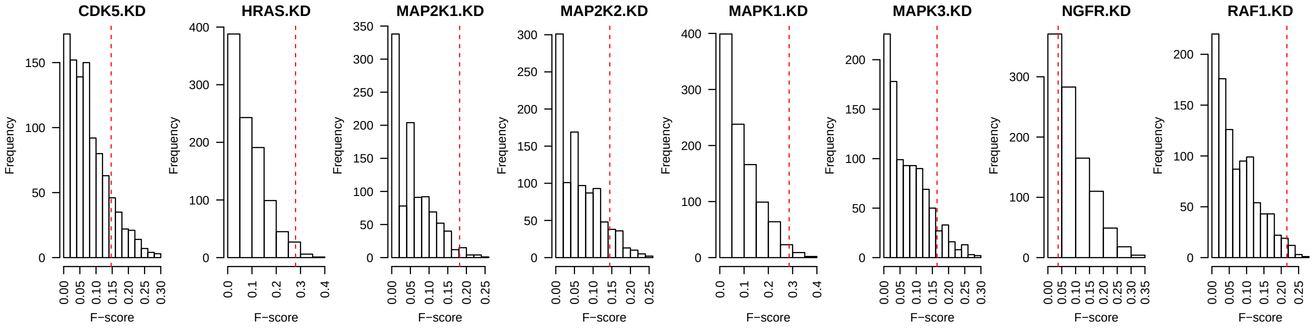

(b) Fscores random expO

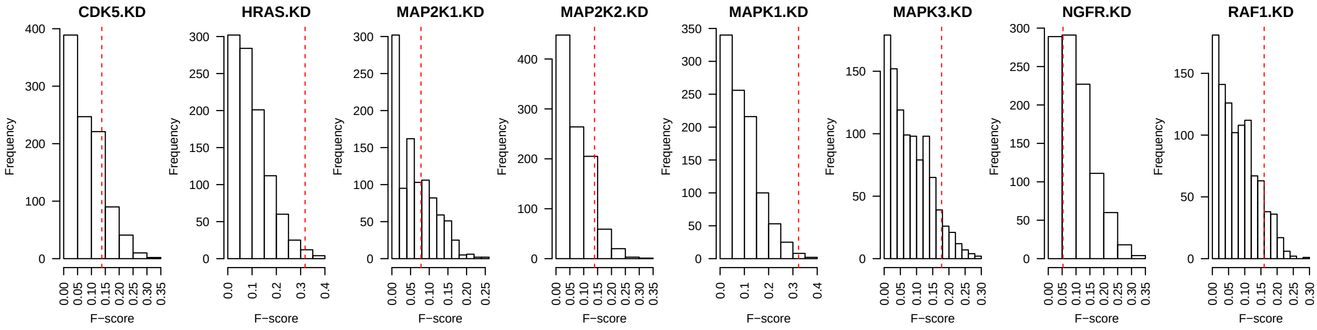

(c) Fscores random jorissen

Figure 5. Each subplot is the histogram of the obtained F-scores for the 1000 random networks. The red line represents the F-score obtained using *predictionet* for the prior source GM5, data set and prior weight $w = 0.5$.

3.6 PRIOR SOURCE: GM6

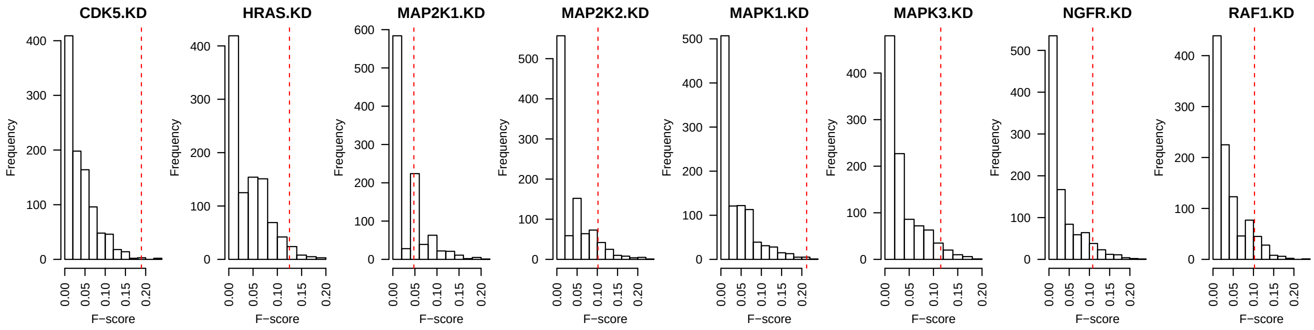

(a) Fscores random KD

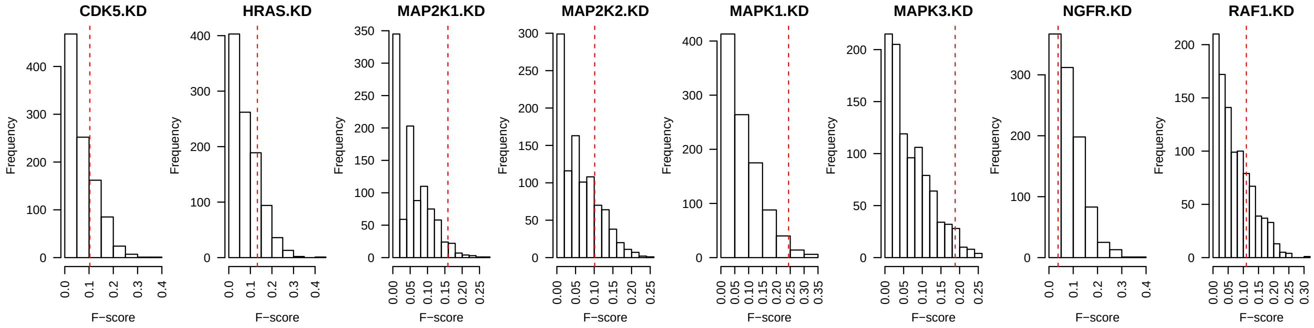

(b) Fscores random expO

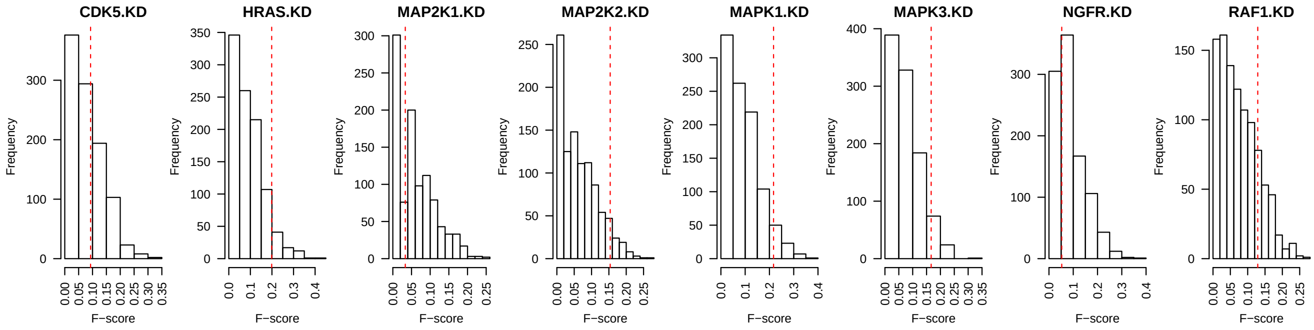

(c) Fscores random jorissen

Figure 6. Each subplot is the histogram of the obtained F-scores for the 1000 random networks. The red line represents the F-score obtained using *predictionet* for the prior source GM6, data set and prior weight $w = 0.5$.

3.7 PRIOR SOURCE: GM7

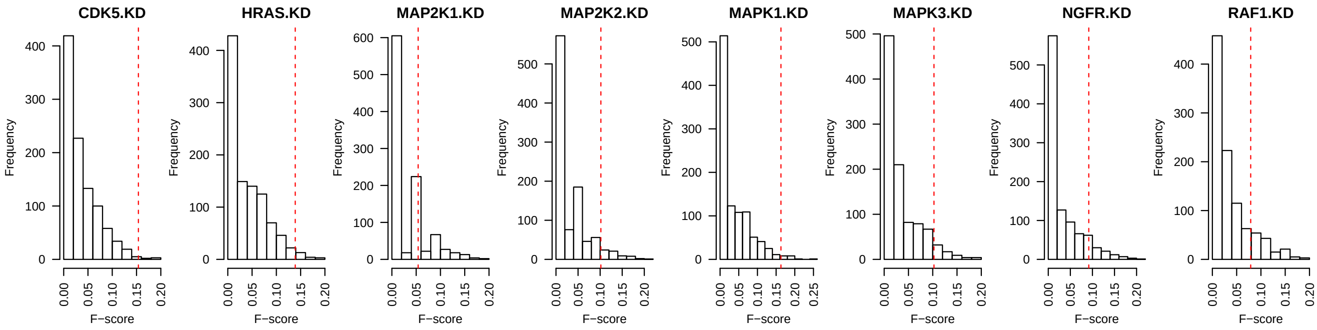

(a) Fscores random KD

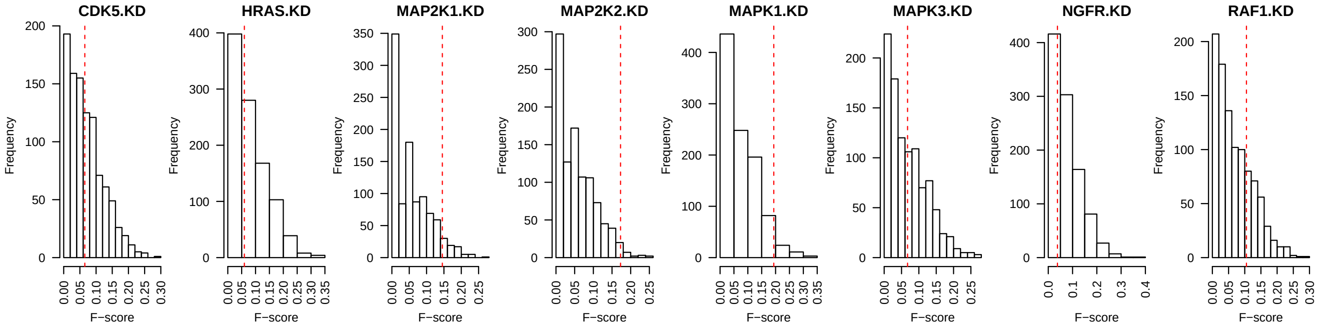

(b) Fscores random expO

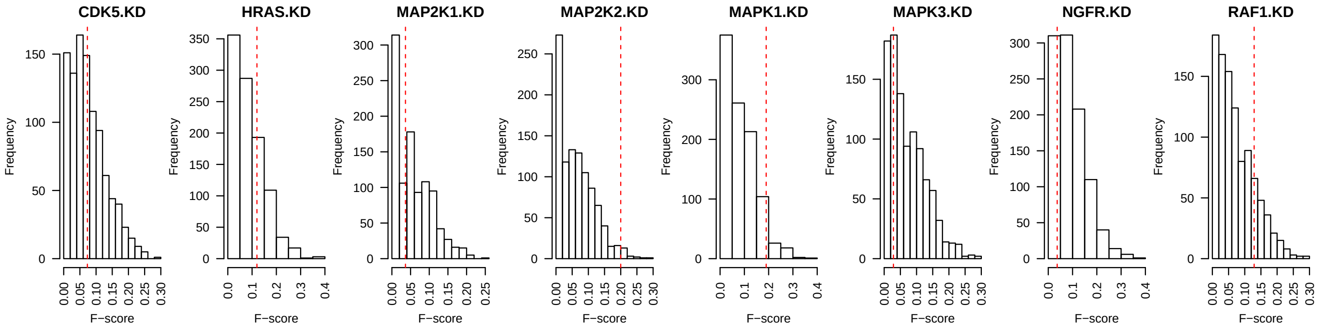

(c) Fscores random jorissen

Figure 7. Each subplot is the histogram of the obtained F-scores for the 1000 random networks. The red line represents the F-score obtained using *predictionet* for the prior source GM7, data set and prior weight $w = 0.5$.

3.8 PRIOR SOURCE: GM8

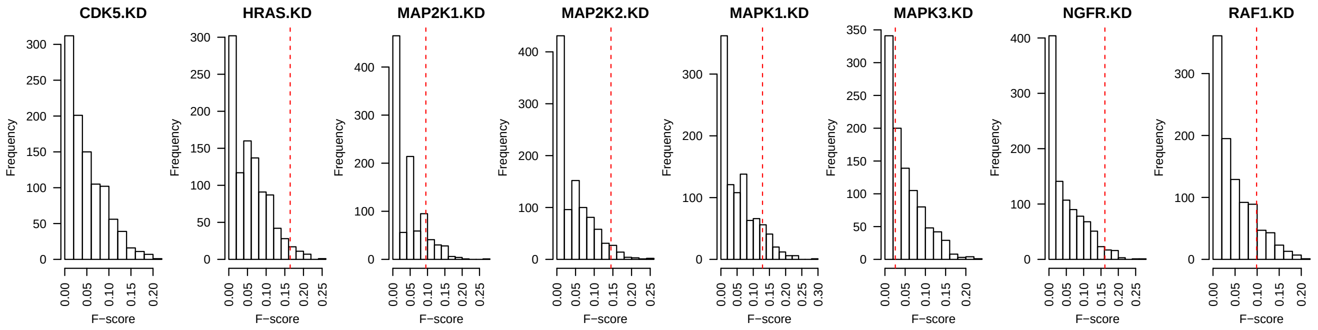

(a) Fscores random KD

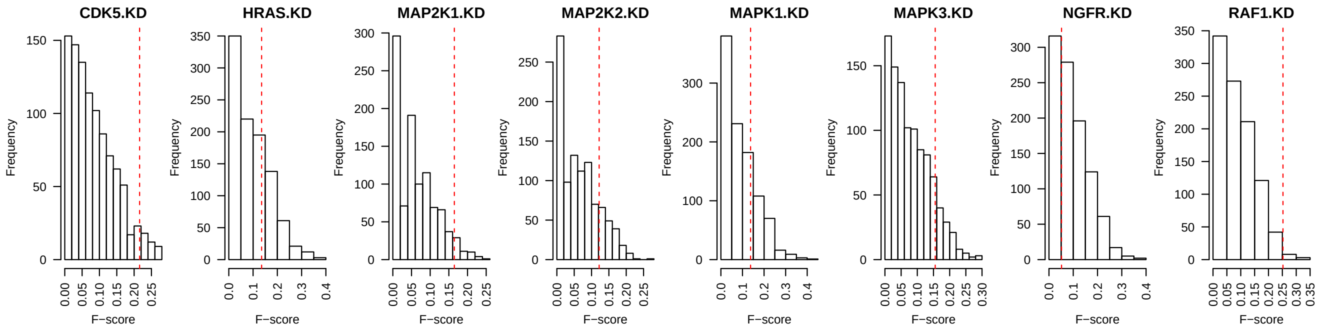

(b) Fscores random expO

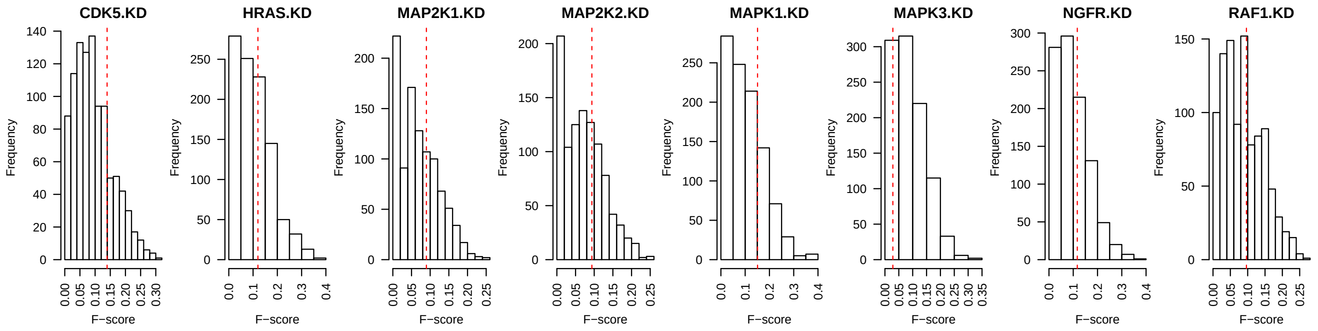

(c) Fscores random jorissen

Figure 8. Each subplot is the histogram of the obtained F-scores for the 1000 random networks. The red line represents the F-score obtained using *predictionet* for the prior source GM8, data set and prior weight $w = 0.5$.

4 COMBINING DATA AND PRIOR SOURCES: HUMAN TUMOR DATA

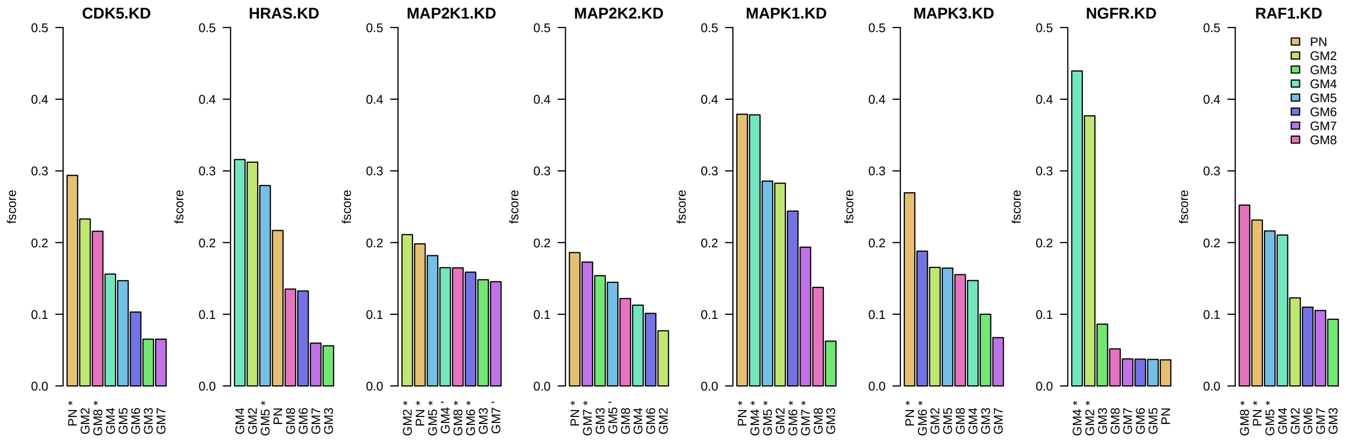

Figure 9. Results when inferring networks with *predictionet* using *expO* data and prior knowledge ($w = 0.5$). The height of each bar corresponds to the obtained F-score, colored by prior source. The x-axis specifes the prior source and includes * if the F-score is significant with p-value < 0.05 and $^-$ for p-values < 0.1.

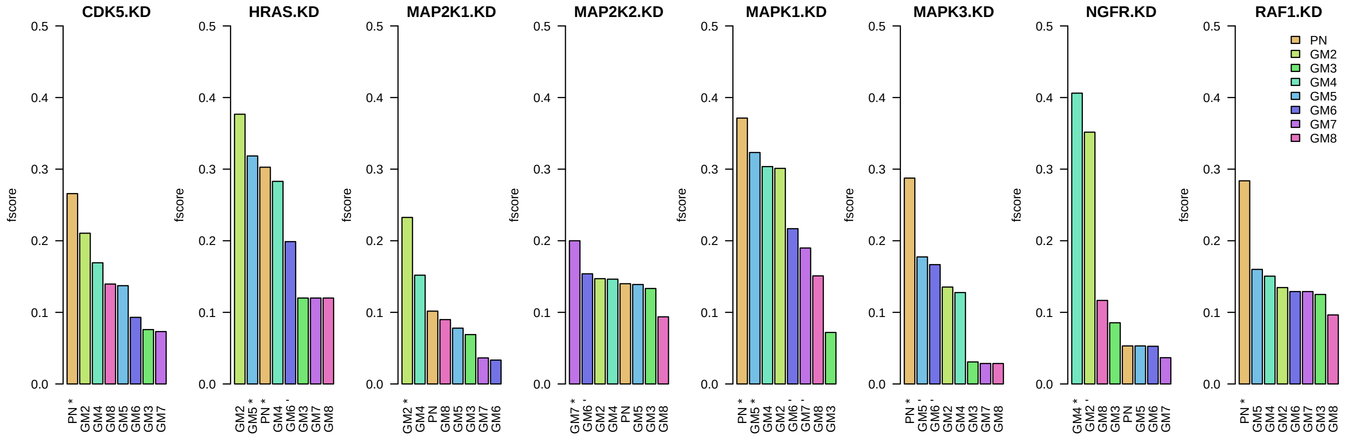

Figure 10. Results when inferring networks with *predictionet* using *jorissen* data and prior knowledge ($w = 0.5$). The height of each bar corresponds to the obtained F-score, colored by prior source. The x-axis specifes the prior source and includes * if the F-score is significant with p-value < 0.05 and $^-$ for p-values < 0.1.

5 COMBINING MULTIPLE PRIOR SOURCES

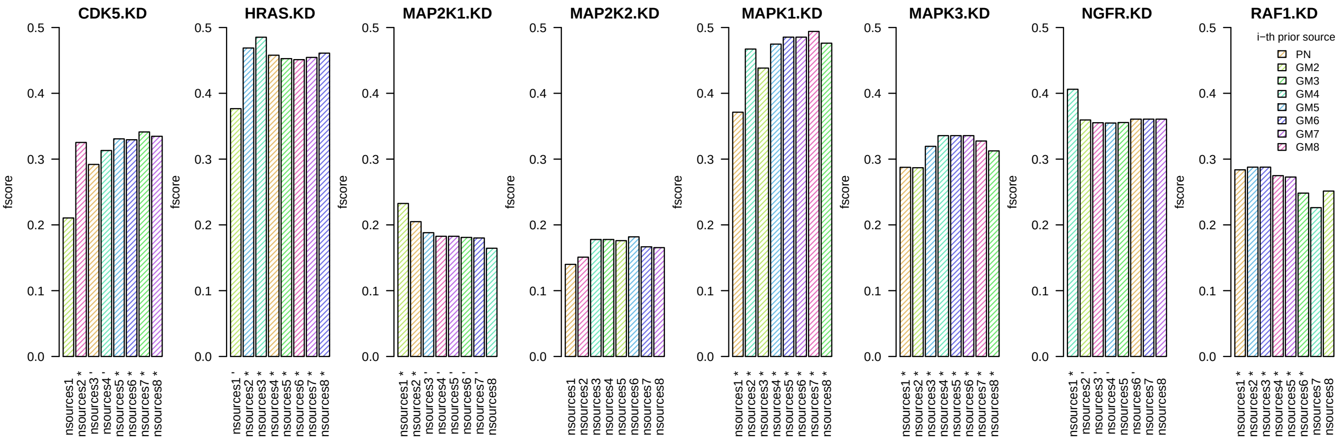

Figure 11. Results when inferring networks with *predictionet* using *jorissen* data and prior knowledge ($w = 0.5$). The height of each bar corresponds to the obtained F-score, colored by which prior source was added. The x-axis specifes the prior source and includes * if the F-score is significant with p-value < 0.05 and $^-$ for p-values < 0.1.
